# Linking first-pass reperfusion success to proteomic markers in large and medium vessel ischemic stroke: an exploratory study

**DOI:** 10.1093/pcmedi/pbag008

**Published:** 2026-02-24

**Authors:** Crhistian-Mario Oblitas, Sabela Fernández-Rodicio, Manuel Rodríguez-Yáñez, Emilio Rodríguez-Castro, Arturo Gonzalez-Quintela, Jacobo Porto-Álvarez, Javier Martínez-Fernández, Miguel Blanco, Jose Luis Taboada, Sara Martnez-Reiriz, Susana B Bravo, Carmen Pena, José Castillo, Maria Luz Alonso-Alonso, Pablo Hervella, Antonio J Mosqueira, Ramón Iglesias-Rey

**Affiliations:** Neuroimaging and Biotechnology Laboratory (NOBEL), Clinical Neurosciences Research Laboratory (LINC), Health Research Institute of Santiago de Compostela (IDIS), Santiago de Compostela 15706, Spain; Neuroimaging and Biotechnology Laboratory (NOBEL), Clinical Neurosciences Research Laboratory (LINC), Health Research Institute of Santiago de Compostela (IDIS), Santiago de Compostela 15706, Spain; Stroke Unit, Department of Neurology, Hospital Clínico Universitario, Santiago de Compostela 15706, Spain; Stroke Unit, Department of Neurology, Hospital Clínico Universitario, Santiago de Compostela 15706, Spain; Department of Internal Medicine, Hospital Clínico Universitario, Santiago de Compostela 15706, Spain; Department of Neuroradiology, Hospital Clínico Universitario, Health Research Institute of Santiago de Compostela (IDIS), Santiago de Compostela 15706, Spain; Department of Neuroradiology, Hospital Clínico Universitario, Health Research Institute of Santiago de Compostela (IDIS), Santiago de Compostela 15706, Spain; Department of Neuroradiology, Hospital Clínico Universitario, Health Research Institute of Santiago de Compostela (IDIS), Santiago de Compostela 15706, Spain; Department of Neuroradiology, Hospital Clínico Universitario, Health Research Institute of Santiago de Compostela (IDIS), Santiago de Compostela 15706, Spain; Department of Neuroradiology, Hospital Clínico Universitario, Health Research Institute of Santiago de Compostela (IDIS), Santiago de Compostela 15706, Spain; Proteomic Unit, Health Research Institute of Santiago de Compostela (IDIS), Complejo Hospitalario Universitario de Santiago de Compostela (CHUS), Santiago de Compostela 15706, Spain; Proteomic Unit, Health Research Institute of Santiago de Compostela (IDIS), Complejo Hospitalario Universitario de Santiago de Compostela (CHUS), Santiago de Compostela 15706, Spain; Neuroimaging and Biotechnology Laboratory (NOBEL), Clinical Neurosciences Research Laboratory (LINC), Health Research Institute of Santiago de Compostela (IDIS), Santiago de Compostela 15706, Spain; Neuroimaging and Biotechnology Laboratory (NOBEL), Clinical Neurosciences Research Laboratory (LINC), Health Research Institute of Santiago de Compostela (IDIS), Santiago de Compostela 15706, Spain; Neuroimaging and Biotechnology Laboratory (NOBEL), Clinical Neurosciences Research Laboratory (LINC), Health Research Institute of Santiago de Compostela (IDIS), Santiago de Compostela 15706, Spain; Neuroimaging and Biotechnology Laboratory (NOBEL), Clinical Neurosciences Research Laboratory (LINC), Health Research Institute of Santiago de Compostela (IDIS), Santiago de Compostela 15706, Spain; Department of Neuroradiology, Hospital Clínico Universitario, Health Research Institute of Santiago de Compostela (IDIS), Santiago de Compostela 15706, Spain; Neuroimaging and Biotechnology Laboratory (NOBEL), Clinical Neurosciences Research Laboratory (LINC), Health Research Institute of Santiago de Compostela (IDIS), Santiago de Compostela 15706, Spain

Dear Editor,

The so-called modified first-pass effect (mFPE) following mechanical thrombectomy, defined as a reperfusion grade with a thrombolysis in cerebral infarction scale ≥ 2b after the first pass of the thrombectomy device, has been proposed as a therapeutic goal to achieve since it has been independently associated with better functional outcomes and lower risk of procedural complications [1–4], establishing mFPE as a crucial indicator of thrombectomy success in patients with acute ischemic stroke (IS). Currently, first-pass reperfusion (FPR) is achieved in only 25% to 35% of patients, underscoring the need for a deeper understanding to improve these rates [5]. Several factors have been associated with FPR outcomes, including patient age, sex, occlusion location, type of anesthesia, and the thrombectomy technique used [6]. Previous studies have explored the relationship between thrombi composition and FPE success using histological techniques, finding that lower red blood cell (RBC) content, higher fibrin levels, and increased neutrophil extracellular traps were associated with a lower likelihood of achieving FPE [7]. This highlights that the structure and composition of thrombi may be relevant and could yield insights to improve understanding of the pathophysiological pathways involved, opening opportunities for novel management approaches.

We conducted an exploratory study to examine the qualitative and quantitative proteomic profiles of thrombi composition in consecutive prospectively recruited patients aged >18 years with acute IS who underwent mechanical thrombectomy, comparing those who achieved an mFPE with those who did not. In this study, mFPE was defined as a reperfusion grade ≥ 2b on the thrombolysis in cerebral infarction scale after the first pass of the thrombectomy device. A qualitative and quantitative protein analysis was performed using LC-MS/MS (Liquid chromatography–mass spectrometry) in data-dependent acquisition mode and SWATH-MS (Sequential Window Acquisition of all Theoretical fragment ion Spectra), respectively. Proteins identified were selected for screening only if the false Discovery Rate (FDR) < 1% at both peptide and protein levels, and if a threshold of a minimum of two peptides per protein was met. Furthermore, dysregulated proteins were considered when the *P*-value for the range test was < 0.05 and the fold-change (FC) was > 1.5 (upregulated) or < 0.6 (downregulated) (see online supplementary material for more details).

A total of 88 consecutive patients were included in this study, with a median age of 75 years (±16 years) and a slight female predominance (55.7%; 49 patients). The most frequent occlusion location was the middle cerebral artery in 88.6% (78 patients), predominantly in the M1 segment (75.6%), and the internal carotid artery in 33% (29 patients). mFPE was observed in 51.1% (45 patients) and was associated with a higher proportion of functional independence at 90 days (62.2% versus 37.8%; *P* = 0.02), but not with overall mortality (45% versus 55%; *P* = 0.69). Supplementary Table 1 (see online supplementary material) shows baseline characteristics.

In the data-dependent acquisition analysis, we used Scaffold to obtain spectral counts, enabling a semiquantitative assessment based on spectral counting. Therefore, the total spectral count was normalized before analysis. A total of 511 unique proteins were observed (8 935 unique peptides and 52 874 unique spectra), of which 502 were common to both groups. Notably, only 9 proteins were differentially expressed (*P* < 0.05 and FC > 1.5) in those patients who achieved mFPE versus those who did not, encoded by genes ACTN1, ACTN4, PPBP, CALR, HSP90AB1, HSP90B1, PDIA3, CLTC, and ANXA1 (supplementary Table 2 and supplementary Fig. 1, see online supplementary material). In the quantitative analysis, SWATH-MS was used to compare protein expression profiles between patients who achieved mFPE and those who did not. A total of 3189 proteins were observed with an FDR < 1%, while only 94 proteins showed a *P* < 0.05. Then, these proteins were evaluated to determine whether they met the cutoffs: FC > 1.5 for upregulated proteins and FC < 0.6 for downregulated proteins. After applying these criteria, a total of 79 distinct proteins were identified among patients who achieved mFPE (supplementary Table 3, see online supplementary material). Nevertheless, for a cutoff log_2_FC of ± 0.5 and a *P* < 0.05, a total of 105 proteins were identified in the statistical analysis (supplementary Table 4, see online supplementary material). A volcano plot for log_2_ of FC showed that 55 proteins were up-regulated, while 50 proteins were down-regulated (Fig. 1A, B), that are involved in different biological pathways related to the coagulation-hemostatic system and immune system, as well as relevant molecular functions such as cadherin and actin binding proteins (supplementary Fig. 2, see online supplementary material). Between the main up-regulated proteins (*P* < 0.05 and FC > 1.5) were found: GP9, HAX1, ITGB3, LGALS1, MT-ND5, POTEF, PPBP, S100A9, SNCG, UBFD1, and UBL4A; while the main down-regulated proteins (*P* < 0.05 and FC < 0.6) found were: ADAP1, AP1G2, CTSA, FTH1, ICAM2, IGHV3-74, KHNYN, PLEKHG5, and RAB27B. Furthermore, the abundance of these 79 dysregulated proteins (50 upregulated and 29 downregulated) was assessed using their SWATH-MS normalized areas, being divided into two groups: those with values above the 75th percentile (high expression) and those below the 25th percentile (low expression). Among the up-regulated proteins with an FC > 2, only POTEF, GP9, ACTBL2, LIN9, ARFIP1, HBA1, and LCN2 were highly expressed, whereas only FANCD2 was lowly expressed (Fig. 1C and D). On the other hand, for the down-regulated group with an FC < 0.5, only PPP1R13L, ATXN1, and POLR2E were highly expressed, whereas KHNYN, ADAP1, and ATP5J2 were lowly expressed (Fig. 1E and F).

**Figure 1:**
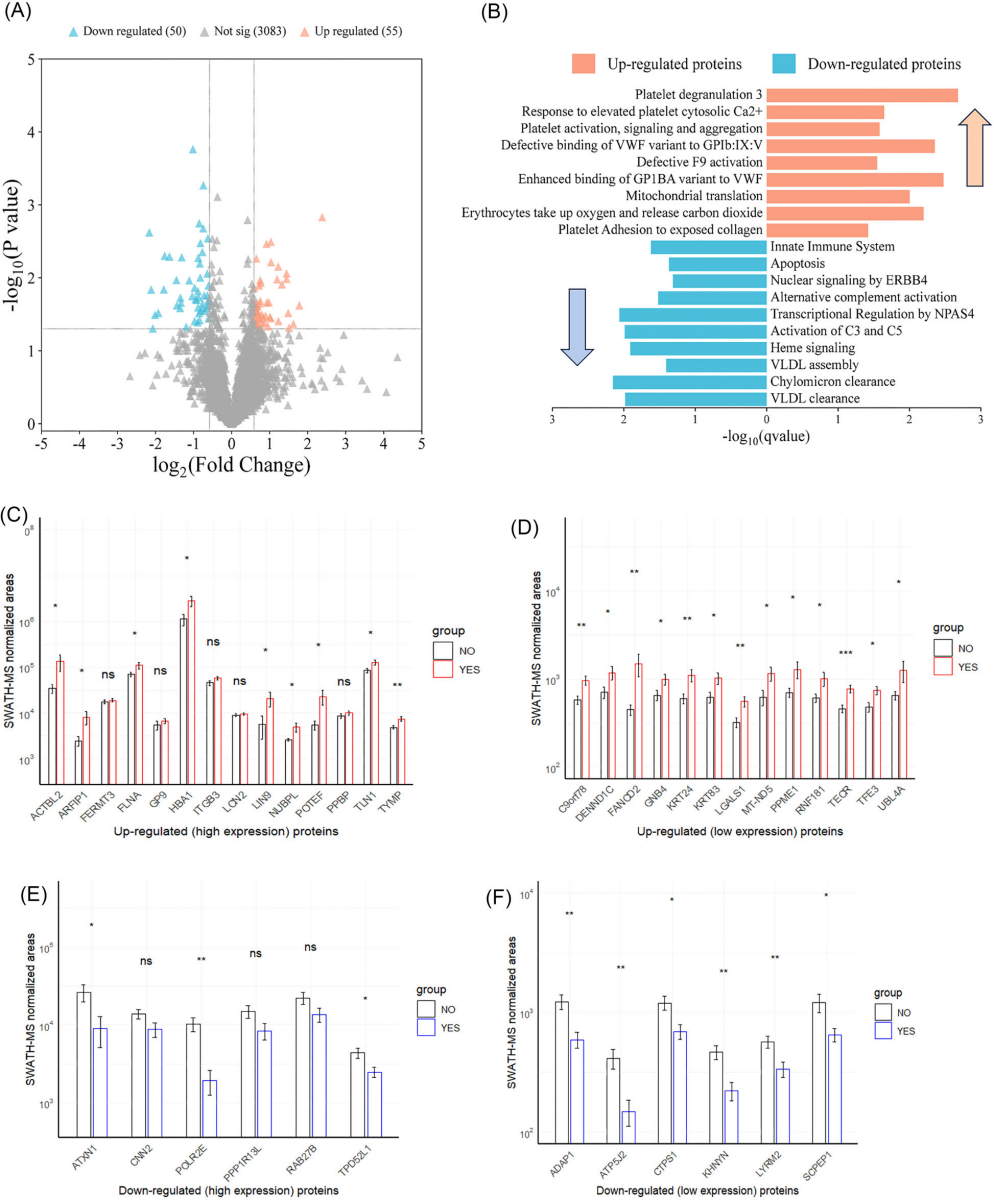
(**A**) Volcano plot for proteins obtained by SWATH-MS, highlighting those that were statistically significant (*P* < 0.05) and a cutoff of ± 0.5 for the log_2_(fold-change). (**B**) Schematization of the functional protein association network (biological processes, cellular components, and molecular functions) for the distinctively dysregulated proteins analyzed by SWATH-MS. Additionally, box plots represent SWATH-MS normalized areas of those proteins with a *P* < 0.05 and a fold-change > 1.5 (upregulated, in red) or < 0.6 (downregulated, in blue), divided into two groups depending on whether they were above the 75th percentile (high expression) (**C, D**) or below the 25th percentile (low expression) (**E, F**). *denotes p <0.05; **denotes p <0.01; ***denotes p <0.001.

Previous work by Vandelanotte *et al*., has suggested that thrombus composition may play a critical role in understanding why patients with similar age, sex, and comorbidities exhibit differing responses in achieving the so-called FPE, which is closely associated with improved functional outcomes. In this setting, as is well established, the time elapsed since IS onset is crucial, where the earlier the intervention the fresher the thrombus, typically characterized by higher RBC content and lower fibrin levels, with changes in the inflammatory environment. Moreover, endogenous factors that regulate blood–brain barrier integrity by inhibiting microglial activation and preventing blood–brain barrier leakage could contribute to neuroinflammation, potentially affecting the achievement of mFPE [7, 8].

This exploratory proteomic study assessed thrombus composition in patients with acute IS undergoing mechanical thrombectomy who achieved the mFPE. The results provide new insights into the biological complexity and molecular signatures associated with this procedural outcome, revealing distinct protein expression patterns related to platelet activation and haemostasis (ACTN1, ACTN4, FLNA, TLN1, ITGB3, PPBP), atherosclerosis and vascular remodelling (SCPEP1, LGALS1, ADAP1, HBA1), and inflammatory and stress-related responses (ANXA1, S100A9, HSP90AB1, HSP90B1). These findings support the utility of thrombus analysis as a platform for future exploration of potential prognostic biomarkers. Additional preclinical and clinical research is needed to elucidate the functions of the dysregulated proteins identified in this exploratory study and to assess their potential clinical significance.

## Supplementary Material

pbag008_Supplemental_File

## Data Availability

The mass spectrometry proteomics data have been deposited to the ProteomeXchange Consortium via the PRIDE [9] partner repository with the dataset identifier PXD066161. The clinical database is not available for legal and ethical reasons.
