# Supplementary material for: Linking first-pass reperfusion success to proteomic markers in large and medium vessel ischemic stroke: an exploratory study
Source: Precis Clin Med. 2026 Feb 24;9(1):pbag008. doi: 10.1093/pcmedi/pbag008 (PMC13006874; doi:10.1093/pcmedi/pbag008)
Supplement: pbag008_Supplemental_File [file pbag008_supplemental_file.docx]

**Supplementary Materials**

**Methods**

***Study design***

We conducted an exploratory study assessing qualitative and quantitative proteomic characterization of thrombi composition in all consecutive prospectively recruited patients older than 18 years with acute IS events who underwent MT, with or without prior intravenous fibrinolysis, at the Hospital Clínico Universitario de Santiago de Compostela (Spain) between January 2024 and December 2024, with a follow-up of 3 months. All these patients were admitted to the stroke unit of the same hospital and treated according to national society guidelines. For recanalization, interventional neuroradiologists employed mechanical thrombectomy with thromboaspiration alone or combined with a stent retriever as the primary technique. The stent retriever alone or combined with thromboaspiration were employed as a second-intention technique. Experienced neuroradiologists conducted the procedures.

All patients underwent cerebral computed tomography (CT) angiography upon admission, followed by cranial CT imaging within 24-48 hours after MT. The exclusion criteria were: 1) absence of CT angiography at admission; 2) MT contraindication at the discretion of the attending physician; 3) absence of written informed consent.

***Clinical variables and Neuroimaging studies***

We included demographic variables, vascular risk factors, comorbidities, time from symptom onset to diagnosis or MT, blood pressure at admission, blood count and biochemical profile at admission, neuroimaging variables, and concomitant reperfusion therapies in the acute phase (intravenous fibrinolysis). Stroke etiology classification was made using the TOAST (Trial of Org 10,172 in Acute Stroke Treatment) criteria^18^. Functional outcomes at 90 days were assessed using the mRankin scale^19^, where functional independence was defined as a mRankin score of 0–2, functional dependence as a score of 3–5, and death as a mRankin score of 6.

Neuroimaging included CT angiography at diagnosis and CT control within 48 hours, according to the local protocol. Neuroimaging evaluations and classification were performed by expert neuroradiologists, blinded to clinical data, concerning to the TICI scale^20^, where poor reperfusion was represented from 0 to 2a grade while good reperfusion with a score of 2b to 3 grade, mFPE^10^ (defined in this study as a TICI scale ≥2b after the first device pass), and the Alberta Stroke Program Early CT Score (ASPECTS) score^21^.

The routine biomarker profile for IS patients at admission was analyzed in the Biochemistry Laboratory of the University Clinical Hospital of Santiago de Compostela.

***Proteomic Analysis for Thrombi Tissue (Qualitative and Quantitative Data)***

All thrombi extracted by MT were stored in phosphate-buffered saline and frozen at −80°C in Corning^TM^ cryotubes until subjected to qualitative (DDA, Data-Dependent Acquisition) and quantitative (SWATH-MS, Sequential Window Acquisition of All Theoretical fragment ions) LC-MS/MS proteomic analyses.

***Protein Extraction and Digestion***

Frozen tissue (100 mg) from the different brain samples was homogenized in 300 µL RIPA buffer [200 mmol/L Tris/HCl (pH 7.4), 130 mmol/L NaCl, 10% (v/v) glycerol, 0.1% (v/v) SDS, 1% (v/v) Triton X-100, and 10 mmol/L MgCl2] with antiproteases and antiphosphatases (Sigma-Aldrich, St. Louis, MO, USA) in a TissueLyser II (Qiagen, Tokyo, Japan). The homogenate was centrifuged at 14,000× g at 4°C for 20 min. The protein concentration was measured using an RC-DC kit (Bio-Rad Laboratories, Hercules, CA, USA) according to the manufacturer’s protocol. Protein aliquots of 100 µg were concentrated in a single SDS-PAGE band^22,23^ and submitted to a manual digestion as previously described^23^. Finally, after peptide extraction using 50% (v/v) ACN/0.1% (v/v) TFA (×3) and ACN (×1), the peptides were pooled, concentrated in a SpeedVac system (Thermo Fisher Scientific, Waltham, MA, USA), and stored at −20°C.

***Qualitative (LC-MS/MS) DDA Analysis***

From each sample, 4 µg of the digested peptides was separated using reverse-phase chromatography for protein identification. The gradient was developed using a micro-LC system (Eksigent Technologies nanoLC 400, Sciex, Redwood City, CA, USA) coupled to a high-speed Triple TOF 6600 mass spectrometer (Sciex, Redwood City, CA, USA) with a microflow source. The analytical column used was a Chrom XP C18 silica-based reversed-phase column (150 0.30 mm) with a 3 mm particle size and 120 Å pore size (Eksigent, Sciex, Redwood City, CA, USA). The trap column was a YMCTRIART C18 (YMC Technologies Teknokroma Analítica, Barcelona, Spain), with a 3 mm particle size and 120 Å pore size, which was switched on-line with the analytical column. Data were acquired using a TripleTOF 6600 system (Sciex, Redwood City, CA, USA) with a data-dependent acquisition (DDA) workflow. The micro-pump generated a flow rate of 5 µL/min and was operated under gradient elution conditions, using 0.1% formic acid in water as mobile phase A and 0.1% formic acid in acetonitrile as mobile phase B. Peptides were separated using a 90 min gradient ranging from 2% to 90% mobile phase B^24^.

Data was acquired using a TripleTOF 6600 System (Sciex, Redwood City, CA, USA) with a DDA workflow. The source and interface conditions were as follows: ion spray floating voltage (ISVF), 5500 V; curtain gas (CUR) 25, collision energy (CE) 10, and ion source gas 1 (GS1) 25. The instrument was operated using Analyst TF 1.7.1 software (Sciex, Redwood City, CA, USA). The switching criteria were set to ions greater than a mass-to-charge ratio (m/z) of 350 and smaller than m/z 1400, with a charge state of 2–5, a mass tolerance of 250 ppm, and an abundance threshold of more than 200 counts per second (cps). The target precursor ions were excluded after 15 s. The instrument was automatically calibrated every 4 hours using tryptic peptides from PepCalMix (Sciex, Redwood City, CA, USA) as an external calibrant^25^.

After the MS/MS analysis (MS2 data), the data files were processed using ProteinPilot 5.0.1 software from Sciex, which uses the algorithm Paragon for database searches and Progroup for data grouping. Data was searched for using the Human-specific UniProt database, specifying iodoacetamide at cysteine alkylation as a variable modification and methionine oxidation as a fixed modification. The false discovery rate (FDR) was determined using a nonlinear fitting method, displaying only the results that reported a 1% global FDR or better^26^. Scaffold (version Scaffold-5.2.2, Proteome Software Inc., Portland, OR, USA) and Scaffold DDA (version Scaffold DDA-6.4.1, Proteome Software Inc., Portland, OR, USA) were used to perform the DDA analysis, as described previously^24^.

***Quantitative Analysis by SWATH-MS method (DIA- Data Independent Acquisition)***

*Generation of Reference Spectral Library*

A pool from each group (mFPE no and mFPE yes) was analyzed using the shotgun DDA approach. The samples were separated in the micro-LC system Ekspert nLC425 (Eksigen, Dublin, CA, USA), using a Chrom XP C18 150 mm × 0.30 mm, 3 mm particle size, and 120 Å pore size (Eksigen, Dublin, CA, USA) at a flow rate of 10 µL/min, using as a solvent A water, 0.1% formic acid (FA), and solvent B acetonitrile (ACN), 0.1% FA. The peptide separation gradient was from 5% to 95% B for 30 min, 90% B for 5 min, and 5% B for 5 min for column equilibration, for a total time of 40 min. A 6600 + hybrid quadrupole-TOF mass spectrometer (Sciex, Redwood City, CA, USA) was coupled with the LC. A 250 ms survey scan was performed from 400 to 1250 m/z using a mass spectrometer, followed by MS/MS experiments from 100 to 1500 m/z (acquisition time of 25 ms) for a total cycle time of 2.8 s. The fragmented precursors were added to the dynamic exclusion list for 15 s, and any ion with a charge +1 was excluded from the MS/MS analysis. Protein identification was performed using ProteinPilot software v.5.0.1. (Sciex, Redwood City, CA, USA) using the Human-specific UniProt database. The FDR was set to 1 for peptides and proteins, with confidence scores above 99%^14,26^.

*Quantification by SWAT-MSH and Data Analysis*

A quantitative proteomic analysis was performed using the SWATH method on a hybrid quadrupole-TOF mass spectrometer, 6600+ (Sciex, Redwood City, CA, USA), as previously described^27,28^. SWATH–MS acquisition was performed using an IDA (independent data analysis) method. Protein from each sample (4 µg) was subjected to chromatographic separation, as described previously. The SWATH–MS method is based on repeating a cycle consisting of the acquisition of 100 TOF MS/MS scans (400–1500 m/z, high-sensitivity mode, 50 ms acquisition time) of overlapping sequential precursor isolation windows of variable width (1 m/z overlap) covering the 400–1250 m/z mass range, with a previous TOF MS scan (400–1500 m/z, 50 ms acquisition time) for each cycle. The total cycle time was 6.3 s. For each sample set, the width of the 100 variable windows was optimized according to the ion density found in the DDA runs using the SWATH–MS variable window calculator worksheet from Sciex.

The targeted data extraction of the fragment ion chromatogram traces from the SWATH–MS runs was performed by PeakView (version 2.2) using the SWATH–MS Acquisition MicroApp (version 2.0). This application processed the data using the spectral library created from the DDA data loaded into it, which comprised individual samples acquired using the SWATH method. Up to ten peptides per protein and seven fragments per peptide were selected based on the signal intensity to obtain the peak areas, and any shared and modified peptides were excluded from processing.

Integrated peak areas (SWATH–MS areas) were directly exported to MarkerView software (Version 1.3) (Sciex, Redwood City, CA, USA) for a relative quantitative analysis. MarkerView uses processing algorithms that accurately identify chromatographic and spectral peaks directly from raw SWATH data. First, the integrated peak areas were normalized using MLR, and an unsupervised multivariate statistical analysis was performed using principal component analysis (PCA) to compare the data across samples after scaling. A Student’s t-test was performed using MarkerView software to compare the samples.

***Statistical analysis and proteomic functional analysis***

This study reported categorical clinical data as proportions and continuous data as mean and one standard deviation (SD) or median and interquartile range (IQR), according to the type of distribution determined by the Kolmogorov-Smirnov test for a sample with the correction of the significance of Lilliefors. The significance of the differences was estimated using the student’s t-test or the Mann-Whitney U test. One-way analysis of variance (ANOVA) was used to compare differences between more than two groups. The qualitative variables were expressed as percentages. All statistical tests were two-tailed, and a *P* <0.05 was considered statistically significant.

For proteomic assessment, two samples were grouped for analysis. First, proteomic data were grouped by mFPE status (mFPE yes vs. mFPE no). A second analysis was carried out for the IS subtypes (cardioembolic, atherothrombotic, and indeterminate). Proteomic statistical analysis was performed in accordance with the nature of the data. For qualitative data, the Scaffold DDA (version Scaffold DDA-6.4.1, Proteome Software Inc., Portland, OR, USA) was used to perform the DDA analysis with a threshold of an FDR<1%; while for quantitative SWATH-MS analysis normality tests (Shapiro-Wilk test) were performed if possible. Proteins with *P* <0.05 and fold-change (FC) cut-off >1.5 or <0.6 were selected. Log2 or Log10 transformation of the FC was used when appropriate. Box plots were generated using individual SWATH–MS area values for each protein and sample. Multiple testing corrections were applied to the results of both DDA and SWATH-MS analyses (Benjamini-Hochberg) to control the false discovery rate (FDR).

The proteomic functional analysis was performed using free software as STRING (version 12.0; https://string-db.org), Metascape (version 3.5; https://metascape.org), Reactome (Version 92.0; <https://reactome.org>) and SRplot (an online free-to-use platform for building data visuals; <https://www.bioinformatics.com.cn/en>). The statistical significance of enrichment was evaluated using the hypergeometric test and the Benjamini-Hochberg multiple-testing adjustment.

IBM SPSS Statistics v.25 for Windows and R Core Team v.4.2.2 (*tidyverse*, *rstatix*, *dplyr*, and *ggpubr* packages) were used for analyses of clinical variables and for graphics, respectively.

**Limitations**

This study presents several limitations. First, although the relatively small sample size may limit the generalizability of the results to other populations, it enabled a detailed, high-resolution proteomic characterization of thrombi from a well-defined, clinically homogeneous cohort. Second, the absence of independent validation methods for the significantly dysregulated proteins, such as ELISA or Western blot, should be acknowledged. Although such validation would strengthen the interpretation of the underlying biological pathways, It falls beyond the scope of this exploratory study and does not diminish the relevance of the proteomic findings presented here. A key technical limitation of SWATH-MS analysis is its dependence on spectral libraries, which fundamentally limits the number of proteins that can be reliably quantified. Furthermore, DDA exhibits a bias toward highly abundant proteins, rendering it particularly vulnerable to the effects of complex samples and broad dynamic ranges in protein expression. Third, further preclinical and interventional studies will be required to elucidate the functional roles of the dysregulated proteins identified in this study and to determine their potential clinical relevance. Nevertheless, the present study also presents strengths. First, the entire sample was consecutively and prospectively recruited in routine clinical practice, thereby limiting potential selection bias. Second, a key technical strength is the implementation of SWATH-MS. This data-independent acquisition technique offers the advantage of combining comprehensive proteomic coverage with high quantitative accuracy, consistency, and robust reproducibility across experiments.

**Supplementary Table1. Baseline characteristics (n = 88 patients) with a follow-up of 90 days.**

|  | | No mFPE  n=43 | mFPE  n=45 | *p* |
| --- | --- | --- | --- | --- |
| Age, years | 75 ±17 | | 76 ±15 | 0.92 |
| Women, % | 51.1 | | 60.5 | 0.38 |
| Hypertension, % | 71.1 | | 65.1 | 0.55 |
| Diabetes, % | 13.3 | | 18.6 | 0.49 |
| LDL-cholesterol, mg/dL | 93 ±32 | | 92 ±29 | 0.69 |
| Previous stroke, % | 4.4 | | 9.3 | 0.21 |
| Previous IHD, % | 14.6 | | 28.4 | 0.68 |
| Atrial fibrillation, % | 57.8 | | 67.4 | 0.35 |
| Blood glucose, mg/dL | 131.5 ±37.8 | | 126.6 ±35.4 | 0.65 |
| NIHSS at admission | 17 [9-21] | | 16 [8-23] | 0.87 |
| ASPECT | 9 [8-10] | | 9 [8-10] | 0.82 |
| Occlusion location, %  ICA (n=29)  M1 (n=59)  M2-3 (n=34)  Tandem (n=39) | 72.4  50.8  64.7  66.7 | | 27.6  49.2  35.3  33.3 | <0.01  0.94  0.04  0.01 |
| TOAST stroke subtype |  | |  | 0.37 |
| Cardioembolic, % | 55.6 | | 60.5 |  |
| Atherothrombotic, % | 15.6 | | 7.0 |  |
| Indeterminate, % | 11.1 | | 14.0 |  |
| Leukoaraiosis, % | 15.6 | | 41.9 | <0.01 |
| Fibrinolysis (iv), % | 31.1 | | 27.9 | 0.74 |
| TICI ≥2b, % | 86.7 | | 100 | 0.04 |
| Stroke recurrence 90 days, % | 2.2 | | 6.9 | 0.28 |
| mRS 0-2 at 90 days, % | 41.2 | | 67.6 | 0.02 |
| Overall mortality, % | 55 | | 45 | 0.69 |

*ASPECT: Alberta Stroke Program Early CT Score; IHD: ischemic heart disease; IQR: interquartile range; mFPE: modified first-pass effect; mRS: modified Rankin scale for functionally independent; NIHSS: National Institutes of Health Stroke Scale. TICI: thrombolysis in cerebral infarction scale.*

**Supplementary Table 2. Significant dysregulated protein mFPE (*yes* vs. *no*) assessed by qualitative analysis LC-MS/MS.**

| **UnitProt code** | **Gen name** | ***P*** | **Log2 FC** |
| --- | --- | --- | --- |
| O43707 | ACTN4 | <0.01 | 0.6695 |
| P12814-3 | ACTN1 | 0.013 | 0.6310 |
| P02775 | PPBP | 0.013 | 1.0154 |
| P27797 | CALR | 0.033 | 0.7629 |
| P08238 | HSP90AB1 | 0.035 | 0.8374 |
| P30101 | PDIA3 | 0.041 | 0.7857 |
| P14625 | HSP90B1 | 0.046 | 0.7490 |
| Q00610-2 | CLTC | 0.046 | 0.9475 |
| P04083 | ANXA1 | 0.048 | 0.8919 |

**Supplementary Table 3. Distinct protein profiling (mFPE yes vs. mFPE no) by SWATH-MS analysis: up-regulated proteins (FC >1.5) and down-regulated proteins (FC <0.6).**

| **UP-regulated proteins** | | | | |
| --- | --- | --- | --- | --- |
| **UnitProt code** | **Gene symbol** | **Description** | ***P*** | **FC** |
| O00165 | HAX1 | HCLS1 associated protein X-1 | <0.01 | 4.49 |
| A5A3E0 | POTEF | POTE ankyrin domain family member F | 0.01 | 4.34 |
| P14770 | GP9 | Glycoprotein IX platelet | 0.05 | 4.21 |
| Q562R1 | ACTBL2 | Actin beta like 2 | 0.03 | 4.04 |
| Q5TKA1 | LIN9 | Lin-9 DREAM muvb core complex component | 0.03 | 3.81 |
| P53367 | ARFIP1 | ARF interacting protein 1 | 0.01 | 3.44 |
| Q9BXW9 | FANCD2 | FA complementation group D2 | <0.01 | 3.39 |
| Q9P2Y4 | ZNF219 | Zinc finger protein 219 | 0.01 | 3.10 |
| P06702 | S100A9 | S100 calcium binding protein A9 | 0.01 | 2.73 |
| Q5JTZ5 | C9orf152 | Chromosome 9 open reading frame 152 | 0.02 | 2.72 |
| Q99717 | SMAD5 | SMAD family member 5 | 0.03 | 2.56 |
| P69905 | HBA1 | Hemoglobin subunit alpha 1 | 0.02 | 2.55 |
| Q16143 | SNCB | Synuclein beta | 0.02 | 2.52 |
| P28676 | GCA | Grancalcin | 0.01 | 2.49 |
| O76070 | SNCG | Synuclein gamma | 0.05 | 2.29 |
| Q96PY6 | NEK1 | NIMA related kinase 1 | 0.01 | 2.16 |
| P80188 | LCN2 | Lipocalin 2 | 0.02 | 2.05 |
| Q9NYI0 | PSD3 | Pleckstrin and Sec7 domain containing 3 | <0.01 | 2.02 |
| P11441 | UBL4A | Ubiquitin like 4A | 0.04 | 1.96 |
| Q96SL4 | GPX7 | Glutathione peroxidase 7 | 0.01 | 1.96 |
| Q92828 | CORO2A | Coronin 2A | 0.02 | 1.93 |
| P03915 | MT-ND5 | NADH dehydrogenase subunit 5 | 0.02 | 1.89 |
| Q53HC5 | KLHL26 | Kelch like family member 26 | 0.04 | 1.86 |
| Q9Y570 | PPME1 | Protein phosphatase methylesterase 1 | 0.03 | 1.85 |
| O14562 | UBFD1 | Ubiquitin family domain containing 1 | 0.02 | 1.85 |
| Q2M2I5 | KRT24 | Keratin 24 | 0.01 | 1.84 |
| P05106 | ITGB3 | Integrin subunit beta 3 | 0.02 | 1.83 |
| Q8TB37 | NUBPL | NUBP iron-sulfur cluster assembly factor (mitochondrial) | 0.03 | 1.81 |
| Q96QF0 | RAB3IP | RAB3A interacting protein | <0.01 | 1.81 |
| Q86VX2 | COMMD7 | COMM domain containing 7 | 0.04 | 1.79 |
| Q8WW33 | GTSF1 | Gametocyte specific factor 1 | 0.01 | 1.79 |
| Q86UX7 | FERMT3 | FERM domain containing kindlin 3 | 0.01 | 1.77 |
| P09382 | LGALS1 | Galectin 1 | 0.00 | 1.77 |
| Q9Y3D5 | MRPS18C | Mitochondrial ribosomal protein S18C | 0.03 | 1.76 |
| P02775 | PPBP | Pro-platelet basic protein | <0.01 | 1.71 |
| Q8IV53 | DENND1C | DENN domain containing 1C | 0.03 | 1.70 |
| Q9P0P0 | RNF181 | Ring finger protein 181 | 0.02 | 1.70 |
| Q9NZ63 | C9orf78 | Chromosome 9 open reading frame 78 | <0.01 | 1.68 |
| Q9NZ01 | TECR | Trans-2,3-enoyl-coa reductase | <0.01 | 1.67 |
| P78385 | KRT83 | Keratin 83 | 0.02 | 1.65 |
| Q6R327 | RICTOR | RPTOR independent companion of MTOR complex 2 | 0.03 | 1.63 |
| P21333 | FLNA | Filamin A | 0.03 | 1.58 |
| P19532 | TFE3 | Transcription factor binding to IGHM enhancer 3 | 0.01 | 1.56 |
| Q9UH62 | ARMCX3 | Armadillo repeat containing X-linked 3 | 0.02 | 1.56 |
| P19971 | TYMP | Thymidine phosphorylase | <0.01 | 1.54 |
| Q9HAV0 | GNB4 | G protein subunit beta 4 | 0.04 | 1.53 |
| Q16853 | AOC3 | Amine oxidase copper containing 3 | 0.01 | 1.52 |
| Q9P281 | BAHCC1 | BAH domain and coiled-coil containing 1 | 0.02 | 1.52 |
| Q9Y490 | TLN1 | Talin 1 | 0.02 | 1.52 |
| Q12923 | PTPN13 | Protein tyrosine phosphatase non-receptor type 13 | 0.02 | 1.52 |
| **DOWN-regulated proteins** | | | | |
| **UnitProt code** | **Gene symbol** | **Description** | ***P*** | **FC** |
| Q99439 | CNN2 | Calponin 2 | 0.05 | 0.60 |
| O94827 | PLEKHG5 | Pleckstrin homology & rhogef domain containing G5 | 0.03 | 0.60 |
| Q9NU23 | LYRM2 | LYR motif containing 2 | 0.01 | 0.60 |
| P17812 | CTPS1 | CTP synthase 1 | 0.02 | 0.59 |
| Q9NPJ6 | MED4 | Mediator complex subunit 4 | 0.01 | 0.59 |
| Q16890 | TPD52L1 | TPD52 like 1 | 0.03 | 0.58 |
| O00194 | RAB27B | RAB27B, member RAS oncogene family | 0.04 | 0.57 |
| Q9Y4P3 | TBL2 | Transducin beta like 2 | 0.04 | 0.56 |
| Q9HB40 | SCPEP1 | Serine carboxypeptidase 1 | 0.04 | 0.55 |
| Q8WTU2 | SSC4D | Scavenger receptor cysteine rich family member with 4 domains | 0.03 | 0.54 |
| O75843 | AP1G2 | Adaptor related protein complex 1 subunit gamma 2 | <0.01 | 0.53 |
| Q9Y240 | CLEC11A | C-type lectin domain containing 11A | 0.02 | 0.53 |
| Q56NI9 | ESCO2 | Establishment of sister chromatid cohesion  N-acetyltransferase 2 | 0.05 | 0.52 |
| P10619 | CTSA | Cathepsin A | 0.02 | 0.51 |
| Q8WUF5 | PPP1R13L | Protein phosphatase 1 regulatory subunit 13 like | 0.03 | 0.50 |
| Q96C19 | EFHD2 | EF-hand domain family member D2 | 0.04 | 0.49 |
| O15037 | KHNYN | KH and NYN domain containing | <0.01 | 0.49 |
| O75689 | ADAP1 | Arfgap with dual PH domains 1 | 0.01 | 0.49 |
| Q96EY4 | TMA16 | Translation machinery associated 16 homolog | 0.01 | 0.44 |
| P80365 | HSD11B2 | Hydroxysteroid 11-beta dehydrogenase 2 | 0.01 | 0.43 |
| P48378 | RFX2 | Regulatory factor X2 | 0.04 | 0.43 |
| Q6P1Q0 | LETMD1 | LETM1 domain containing 1 | 0.01 | 0.40 |
| Q8ND56 | LSM14A | LSM14A mRNA processing body assembly factor | 0.01 | 0.37 |
| P56134 | ATP5J2 | ATP synthase membrane subunit f | 0.01 | 0.37 |
| P13598 | ICAM2 | Intercellular adhesion molecule 2 | 0.03 | 0.36 |
| P54253 | ATXN1 | Ataxin 1 | 0.05 | 0.35 |
| P02794 | FTH1 | Ferritin heavy chain 1 | 0.04 | 0.32 |
| A0A0B4J1X5 | IGHV3-74 | Immunoglobulin heavy variable 3-74 | 0.02 | 0.29 |
| P19388 | POLR2E | RNA polymerase II, I and III subunit E | <0.01 | 0.19 |
| *FC denotes fold change; mFPE denotes modified first pass effect.* | | | | |

**Supplementary Table 4. Significant dysregulated protein mFPE (*yes* vs. *no*) assessed by quantitative analysis SWATH-MS.**

| **UnitProt code** | **Gen name** | ***P*** | **Fold Change** | **Log2 FC** |
| --- | --- | --- | --- | --- |
| P69905 | HBA1 | 0.019 | 2.545 | 1.348 |
| P09382 | LGALS1 | 0.003 | 1.766 | 0.820 |
| P02775 | PPBP | 0.004 | 1.705 | 0.770 |
| P78385 | KRT83 | 0.017 | 1.651 | 0.723 |
| P21333 | FLNA | 0.026 | 1.579 | 0.659 |
| P19971 | TYMP | 0.003 | 1.543 | 0.625 |
| Q16853 | AOC3 | 0.013 | 1.523 | 0.607 |
| Q9Y490 | TLN1 | 0.021 | 1.522 | 0.606 |
| O00165 | HAX1 | 0.002 | 4.485 | 2.165 |
| A5A3E0 | POTEF | 0.015 | 4.336 | 2.116 |
| P14770 | GP9 | 0.049 | 4.206 | 2.073 |
| Q562R1 | ACTBL2 | 0.032 | 4.043 | 2.016 |
| Q5TKA1 | LIN9 | 0.030 | 3.806 | 1.928 |
| P53367 | ARFIP1 | 0.014 | 3.442 | 1.783 |
| Q9BXW9 | FANCD2 | 0.005 | 3.389 | 1.761 |
| Q9P2Y4 | ZNF219 | 0.005 | 3.104 | 1.634 |
| P06702 | S100A9 | 0.011 | 2.734 | 1.451 |
| Q5JTZ5 | C9orf152 | 0.024 | 2.717 | 1.442 |
| Q99717 | SMAD5 | 0.026 | 2.558 | 1.355 |
| Q16143 | SNCB | 0.022 | 2.521 | 1.334 |
| P28676 | GCA | 0.005 | 2.485 | 1.313 |
| O76070 | SNCG | 0.047 | 2.289 | 1.195 |
| Q96PY6 | NEK1 | 0.011 | 2.165 | 1.114 |
| P80188 | LCN2 | 0.018 | 2.051 | 1.037 |
| Q9NYI0 | PSD3 | 0.000 | 2.024 | 1.018 |
| P11441 | UBL4A | 0.039 | 1.964 | 0.974 |
| Q96SL4 | GPX7 | 0.014 | 1.963 | 0.973 |
| Q92828 | CORO2A | 0.020 | 1.932 | 0.950 |
| P03915 | MT-ND5 | 0.020 | 1.894 | 0.921 |
| Q53HC5 | KLHL26 | 0.041 | 1.859 | 0.894 |
| Q9Y570 | PPME1 | 0.026 | 1.846 | 0.885 |
| O14562 | UBFD1 | 0.015 | 1.845 | 0.884 |
| Q2M2I5 | KRT24 | 0.005 | 1.835 | 0.876 |
| P05106 | ITGB3 | 0.018 | 1.826 | 0.869 |
| Q8TB37 | NUBPL | 0.030 | 1.810 | 0.856 |
| Q96QF0 | RAB3IP | 0.002 | 1.806 | 0.853 |
| Q86VX2 | COMMD7 | 0.038 | 1.792 | 0.842 |
| Q8WW33 | GTSF1 | 0.006 | 1.785 | 0.836 |
| Q86UX7 | FERMT3 | 0.010 | 1.772 | 0.826 |
| Q9Y3D5 | MRPS18C | 0.028 | 1.762 | 0.817 |
| Q8IV53 | DENND1C | 0.026 | 1.702 | 0.767 |
| Q9P0P0 | RNF181 | 0.023 | 1.698 | 0.763 |
| Q9NZ63 | C9orf78 | 0.002 | 1.679 | 0.748 |
| Q9NZ01 | TECR | 0.001 | 1.675 | 0.744 |
| Q6R327 | RICTOR | 0.028 | 1.633 | 0.708 |
| P19532 | TFE3 | 0.009 | 1.559 | 0.641 |
| Q9UH62 | ARMCX3 | 0.017 | 1.556 | 0.638 |
| Q9HAV0 | GNB4 | 0.036 | 1.526 | 0.610 |
| Q9P281 | BAHCC1 | 0.024 | 1.522 | 0.606 |
| Q12923 | PTPN13 | 0.020 | 1.521 | 0.605 |
| Q99439 | CNN2 | 0.047 | 0.599 | -0.738 |
| P02794 | FTH1 | 0.043 | 0.325 | -1.623 |
| A0A0B4J1X5 | IGHV3-74 | 0.024 | 0.291 | -1.779 |
| O94827 | PLEKHG5 | 0.033 | 0.598 | -0.743 |
| Q9NU23 | LYRM2 | 0.011 | 0.595 | -0.748 |
| P17812 | CTPS1 | 0.021 | 0.593 | -0.755 |
| Q9NPJ6 | MED4 | 0.012 | 0.587 | -0.768 |
| Q16890 | TPD52L1 | 0.032 | 0.577 | -0.794 |
| O00194 | RAB27B | 0.042 | 0.573 | -0.804 |
| Q9Y4P3 | TBL2 | 0.035 | 0.564 | -0.825 |
| Q9HB40 | SCPEP1 | 0.041 | 0.549 | -0.865 |
| Q8WTU2 | SSC4D | 0.033 | 0.539 | -0.891 |
| O75843 | AP1G2 | 0.003 | 0.530 | -0.916 |
| Q9Y240 | CLEC11A | 0.022 | 0.528 | -0.923 |
| Q56NI9 | ESCO2 | 0.046 | 0.516 | -0.955 |
| P10619 | CTSA | 0.022 | 0.508 | -0.976 |
| Q8WUF5 | PPP1R13L | 0.034 | 0.499 | -1.003 |
| Q96C19 | EFHD2 | 0.036 | 0.490 | -1.029 |
| O15037 | KHNYN | 0.003 | 0.489 | -1.032 |
| O75689 | ADAP1 | 0.006 | 0.488 | -1.034 |
| Q96EY4 | TMA16 | 0.010 | 0.437 | -1.195 |
| P80365 | HSD11B2 | 0.007 | 0.428 | -1.224 |
| P48378 | RFX2 | 0.039 | 0.426 | -1.231 |
| Q6P1Q0 | LETMD1 | 0.012 | 0.400 | -1.321 |
| Q8ND56 | LSM14A | 0.010 | 0.368 | -1.443 |
| P56134 | ATP5J2 | 0.009 | 0.368 | -1.443 |
| P13598 | ICAM2 | 0.030 | 0.357 | -1.487 |
| P54253 | ATXN1 | 0.049 | 0.351 | -1.509 |
| P19388 | POLR2E | 0.001 | 0.192 | -2.379 |

**
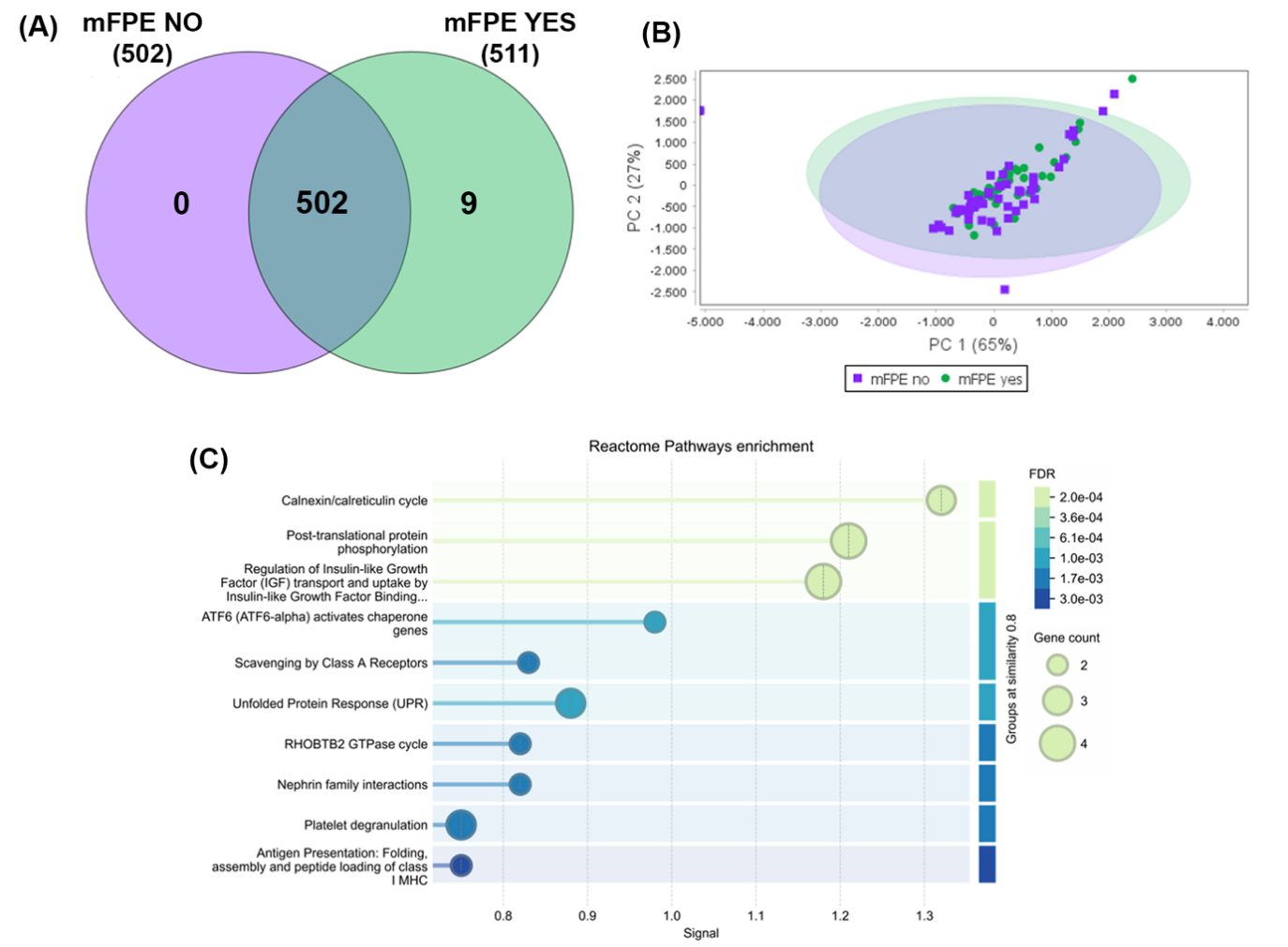
Supplementary Figure 1.** Qualitative LC-MS/MS for the data-dependent acquisition (DDA) analysis. (**A**) Overlapping and distinct proteins with FDR <1% from qualitative analysis are represented in a Venn diagram among patients who achieve the modified first-pass effect (mFPE) or do not (*P* <0.05). (**B**) Principal components analysis (PCA), showing that principal components 1 (PC1) and 2 (PC2) were identified by variance in the DDA analysis. The percentage of variance indicates how much variance was explained by PC1 and PC2. (**C**) Reactome pathways enrichment, in which the 9 distinct proteins were involved between patients who achieved mFPE and those who did not. Panels A and B were obtained from Scaffold DDA software v.6.4.1, while panel C was obtained by STRING (version 12.0; <https://string-db.org>).

**
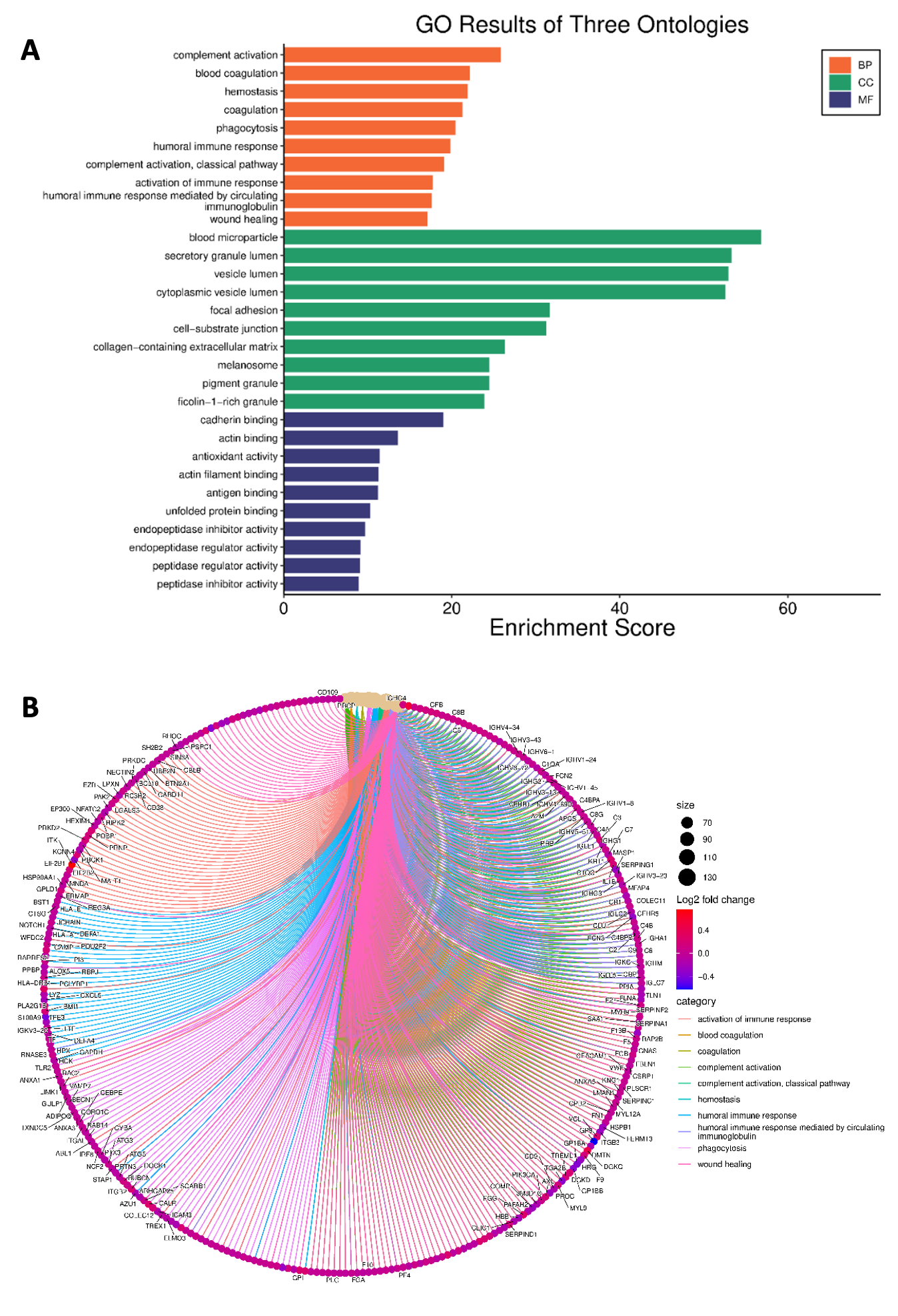
Supplementary Figure 2. (A)** Two-sided plot representing the main dysregulated significant proteins with a fold change >1.5 (upregulated proteins) or <0.6 (downregulated proteins). **(B) Circular network plot showing gene pathways that may be involved in immune and thrombosis processes, visualizing** genes (outer circle) and their associations with different biological processes (center connections). Gene nodes are colored by Log2(fold change), from downregulated (blue) to upregulated (red), as indicated by the color scale. Node size reflects expression levels, with colored links indicating functional categories such as immune response, blood coagulation, complement activation, phagocytosis, and vascular healing, among others.
